# Recent advances and prospects of nanoparticle-based drug delivery for diabetic ocular complications

**DOI:** 10.7150/thno.108691

**Published:** 2025-02-25

**Authors:** Siqi Wang, Hongyu Yang, Jiaying Zheng, Aiyang Tong, Sen Mu, Dongkai Wang, Ming Zhao, Ji Li

**Affiliations:** Department of Pharmaceutics, School of Pharmacy, Shenyang Pharmaceutical University, No. 103, Wenhua Road, Shenyang, 110016, P. R. China.

**Keywords:** diabetes eye complications, nanoparticles, therapeutic approaches, drug delivery, nanotechnology, ophthalmic vehicles

## Abstract

Diabetes mellitus (DM) is a chronic metabolic disorder that significantly affects various organ systems. The systemic effects of DM lead to numerous complications, with ocular manifestations being of particular concern due to their severity and impact on quality of life. Hyperglycemia-induced ocular damage often results in a range of lesions, including diabetic retinopathy (DR), keratopathy, cataracts, and glaucoma. These conditions impose considerable physical discomfort on patients and place a substantial economic burden on healthcare systems. The advent of nanotechnology has facilitated the development of innovative therapeutic strategies for managing diabetic ocular complications. This review highlights several common ocular complications associated with DM, focusing on their pathogenesis and treatment strategies. Emphasis is placed on the innovative applications and potential of nanotechnology in treating diabetic ocular complications.

## 1. Introduction

**Figure 1:**
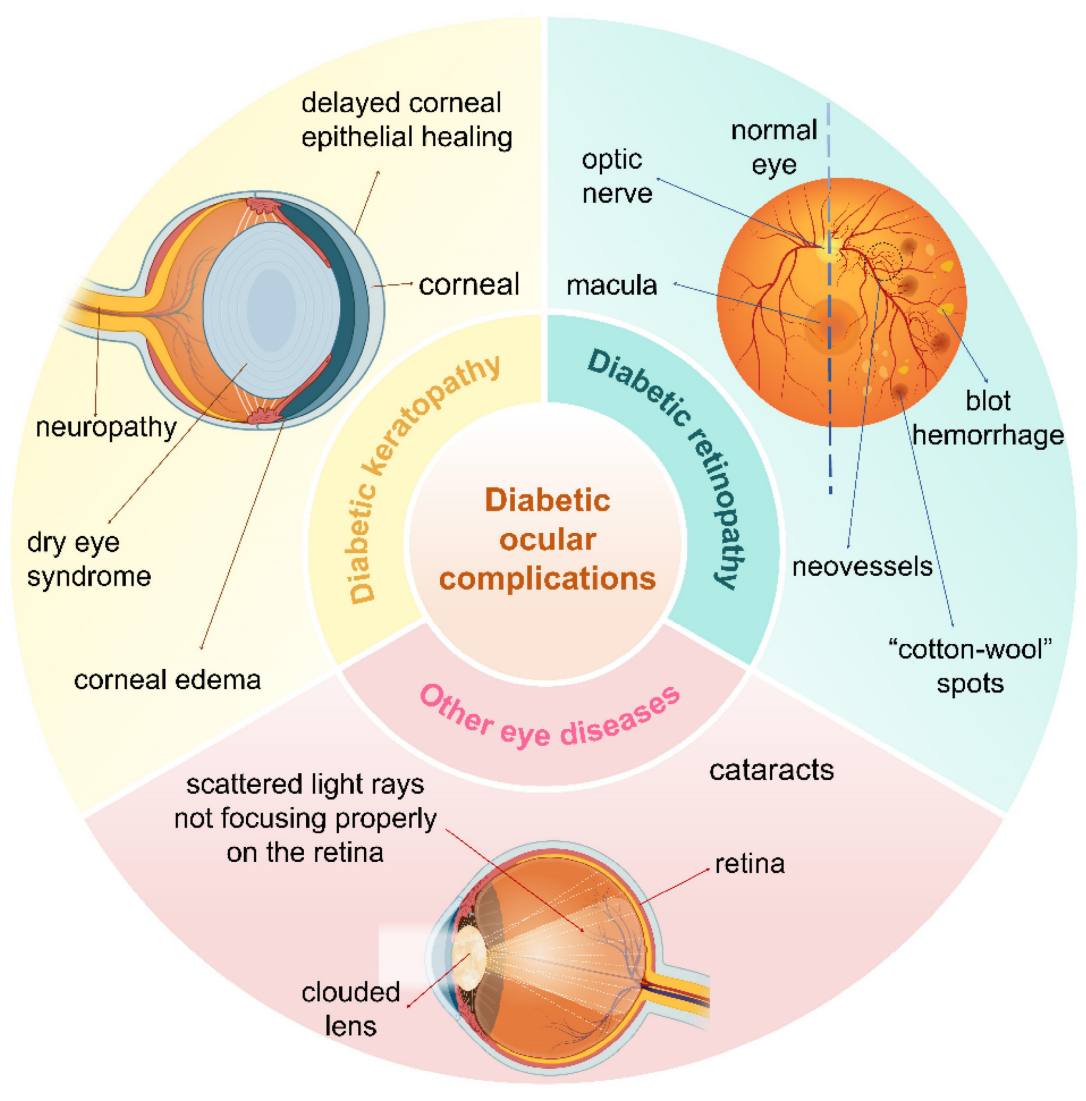
Main types of ocular complications of DM, including diabetic keratopathy, DR, and cataracts. Created with BioRender.com. (http://biorender.com).

The eye is a vital organ in the human body. Diabetic eye complications primarily result from chronic hyperglycemia, which affects multiple ocular structures and contributes significantly to various eye diseases 1. The increasing prevalence of diabetes mellitus (DM) has led to a rise in the incidence of ocular complications. Prominent among these complications are diabetic keratopathy, diabetic retinopathy (DR), cataracts, and glaucoma. These ocular manifestations cause considerable physical discomfort for affected individuals and impose a substantial economic burden on patients and healthcare systems **(Figure 1)**.

**Figure 2:**
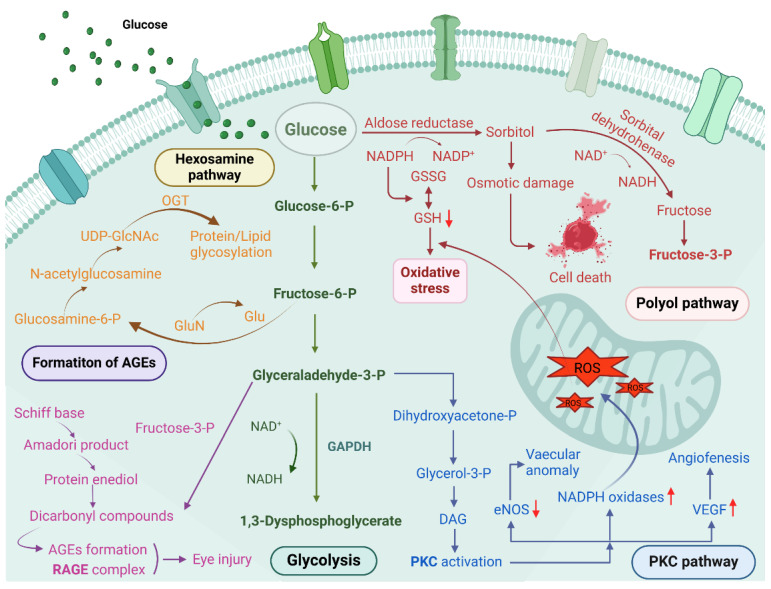
The main pathways causing hyperglycemia include the polyol pathway, hexosamine pathway, PKC activation, and formation of AGEs, etc. Created with BioRender.com. (http://biorender.com).

Following extensive research over the years, significant progress has been made in understanding the pathogenesis of diabetic ocular diseases; however, the mechanisms underlying diabetic dry eye and other ocular lesions remain incompletely understood, which hampers the development of effective clinical treatment strategies. Several common pathogenic mechanisms of hyperglycemia have been proposed, such as increased polyol pathway flux, overactivation of the hexosamine pathway, accumulation of intracellular advanced glycation end products (AGEs), activation of the protein kinase C (PKC) pathway, inflammatory responses, and oxidative stress 2, 3. These mechanisms are summarized below. Since the proposal of a unified mechanism for diabetic complications, growing evidence indicates that reactive oxygen species (ROS) activate multiple signaling pathways, with oxidative stress induced by ROS being a key pathogenic factor in DM and its complications 4 **(Figure 2)**.

The most commonly employed treatment approaches can be categorized into three types 5, 6: (1) Topical administration, primarily for anterior segment diseases; (2) Intraocular administration, which provides superior efficacy compared to topical treatments; and (3) Oral administration, notable for its high patient compliance. These traditional drug delivery systems are cost-effective, convenient, and generally safe. However, the eye's complex physiological barriers and anatomical structures hinder the entry and penetration of drugs into intraocular tissues, often resulting in suboptimal therapeutic outcomes for ocular drug delivery. Tear turnover, blinking, and nasolacrimal drainage rapidly eliminate many topical eye drops 7. Additionally, both static and dynamic barriers restrict the delivery of intraocular medications 8. Oral administration is limited by the blood-retinal barrier, which prevents the influx of molecules towards the vitreous from the bloodstream, regulated by the retinal pigment epithelium 9. Systemic administration typically requires high doses to achieve therapeutic efficacy in the eye 10.

In recent years, nano-delivery systems have made significant progress and hold promise for addressing the challenges of ocular disease treatment. Various carriers, such as liposomes, micelles, lipid nanoparticles, and dendritic macromolecules, have been extensively studied, providing several advantages for ocular drug delivery. These systems extend the residence time and reduce dosing frequency, allowing therapeutic agents to remain in the eye for longer periods. They also enhance drug solubility, thereby improving the bioavailability of therapeutic agents 11. Additionally, these systems promote adhesion and rapid internalization, enhancing their interaction with the ocular surface. In gene therapy, these systems improve both the efficiency and duration of gene transfection, offering a promising approach for treating genetic disorders of the eye and potentially resulting in long-lasting therapeutic effects and improved patient outcomes. Beyond therapy, these systems also enhance ocular diagnosis and imaging, improving diagnostic accuracy and effectiveness. Finally, they can facilitate retinal repair, thereby providing a pivotal benefit in the management of ocular diseases 12.

This paper seeks to increase awareness of the detrimental effects of DM on ocular health and to highlight the ophthalmic complications that arise from the disease, simultaneously elucidating the potential role of nanotechnological interventions in the therapeutic management and monitoring of these conditions. The impact of various ocular complications on the patient and the advantages and limitations of currently available treatments are analyzed. The paper concludes with an exploration of recent advancements in nanotechnology as applied to ocular drug delivery, gene therapy, and other key areas, including diagnostics, sensing, and imaging.

## 2. Diabetic eye complications: addressing major vision risks

The human eye is a complex and vital organ, essential for visual perception. Structurally, the eye is divided into two primary segments: the anterior and posterior segments, each serving distinct functions related to visual processing and ocular protection 13. The anterior segment, consisting of the cornea, iris, lens, and aqueous humor, plays a pivotal role in the eye's anterior functions. The cornea, a refractive tissue composed of five distinct layers (epithelium, Bowman's membrane, stroma, Descemet's membrane, and endothelium), contains an epithelial layer that serves as a formidable barrier through tight junctions, preventing foreign substances from penetrating the eye 14. The posterior segment, comprising the vitreous body, retina, choroid, and posterior sclera, is equally crucial for visual function. The retina, a neural layer, is responsible for converting light signals into electrical impulses. The choroid, abundant in the vasculature, supplies the outer retina and plays a crucial role in regulating the intraocular environment. The sclera provides structural support and is integral to ocular defense mechanisms. However, DM induces progressive, vision-threatening changes in various ocular tissues. Therefore, timely recognition and management of diabetic ocular complications are essential for preserving vision and reducing long-term morbidity. The therapeutic strategies for several common ocular complications are discussed in the following sections.

### 2.1 Approaches for managing diabetic keratopathy

Approximately 47-64% of diabetic patients are affected by diabetic keratopathy 15. Diabetic keratopathy is a prevalent complication in both type 1 and type 2 diabetes mellitus (T1DM and T2DM). As our understanding of diabetic ocular complications improves, and with the rising number of cases involving vision impairment due to corneal lesions, diabetic corneal complications are receiving greater attention in clinical practice 16. Studies have demonstrated that diabetic corneas are exhibit greater susceptibility to bacterial and viral infections than healthy corneas, due to an imbalance in the ocular surface environment 17. DM can impair the corneal structure, including the epithelium, stroma, and endothelium 18.

**Figure 3:**
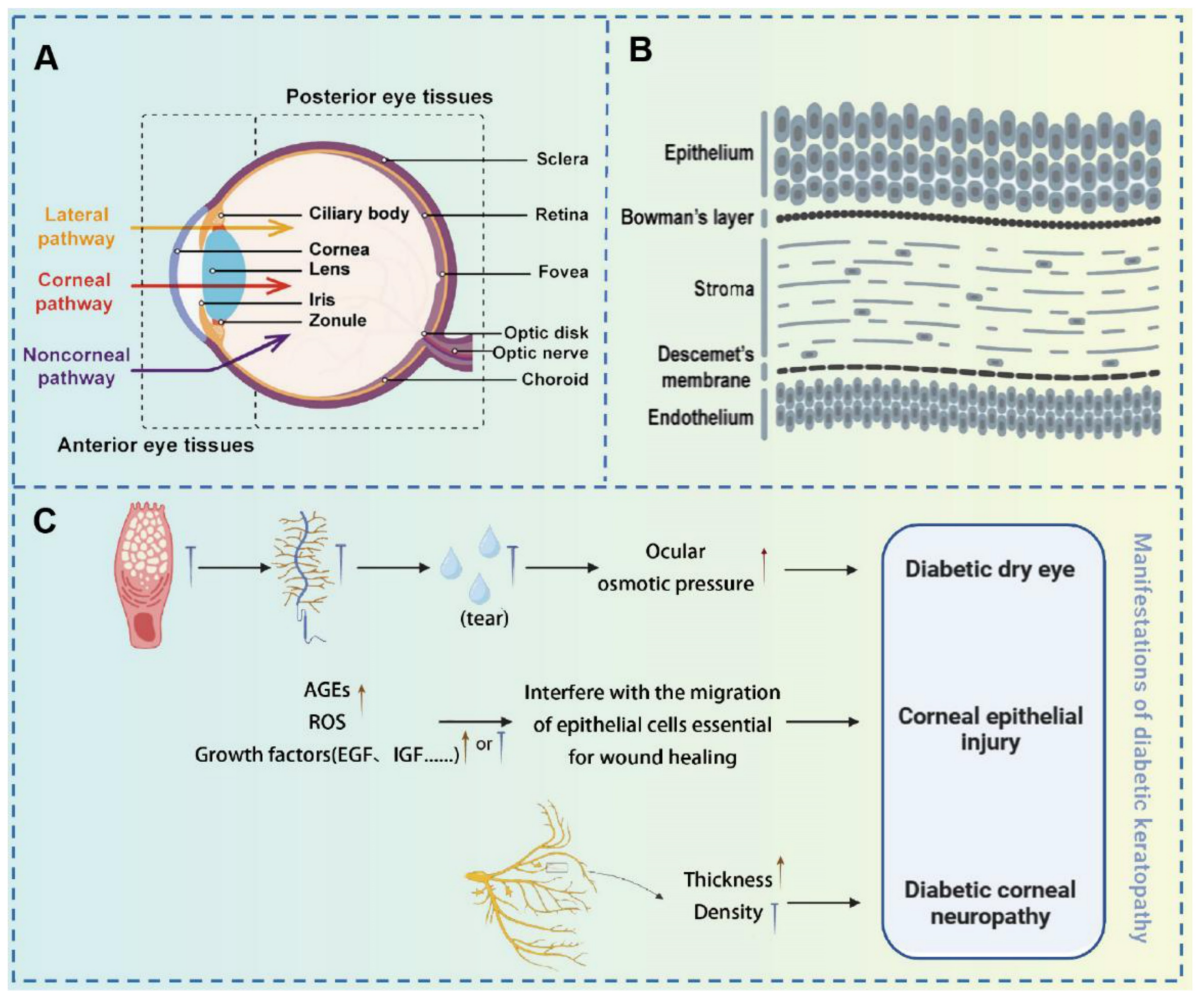
(A) Diagram of ocular anatomical components; (B) Schematic representation of the corneal layers; (C) Manifestations of diabetic keratopathy. **(A-B)** Adapted with permission from 22, copyright 2024, Elsevier. **(C)** Original figure by Siqi Wang.

Hyperglycemia-induced oxidative stress can damage the mitochondria of corneal epithelial cells, reducing available cellular energy, which in turn impairs the cells' proliferation and migration abilities, ultimately leading to delayed corneal epithelial healing 19. Additionally, the accumulation of AGEs can disrupt the homeostasis necessary for corneal epithelial wound healing, further exacerbating corneal epithelial damage 20. Corneal neuropathy is another significant manifestation of diabetic corneal complications. In a hyperglycemic environment, corneal nerve fiber bundles thicken and undergo morphological changes, nerve conduction is impaired, and the concentration of neurotrophic factors decreases, leading to reduced corneal sensation and symptoms such as dry eye disease (DED) 21. These pathological changes not only impair corneal function but also increase the risk of corneal infections and other complications, thereby posing a significant threat to patients' vision **(Figure 3)**.

The previously mentioned symptoms of corneal lesions significantly affect ocular health and present a considerable threat to patients' quality of life. Furthermore, structural, metabolic, and functional changes in the cornea induced by DM pose substantial challenges in management and treatment. Glycemic control remains the primary strategy for preventing the rapid progression of ocular structural damage. Currently, numerous pharmacological agents, including oral medications (e.g., metformin) and injectable therapies (e.g., insulin), are utilized to manage glycemia. However, due to the delay between the onset of DM symptoms and the achievement of effective glycemic control, ocular damage may already be evident by the time treatment begins. This highlights the importance of early diagnosis in preventing the onset of DM-associated ocular complications 5.

Therefore, to prevent or delay ocular damage induced by hyperglycemia, considerable research has focused on developing more effective ophthalmic drugs and optimized drug delivery systems for managing diabetic ocular surface diseases. Current therapeutic strategies include conventional approaches (e.g., topical lubricants, soft bandage contact lenses, and corneal transplantation), antioxidants, growth factors, gene therapy, and novel interventions such as naltrexone therapy. The following table provides a comprehensive analysis of current clinical and experimental treatments for diabetic keratopathy, offering insights into their mechanisms of action, therapeutic efficacy, and potential limitations **(Table 1).**

### 2.2 Therapeutic strategies for DR

Under the prolonged effects of DM, individuals with either T1DM or T2DM are at significant risk of developing DR, a debilitating eye condition that poses a major threat to vision 54. Angiogenesis plays a detrimental role during the progression of DR. Abnormal angiogenesis promotes the progression of non-proliferative diabetic retinopathy (NPDR) to more advanced stages. Although the initial stage of NPDR may be asymptomatic, angiogenesis-related changes are already occurring. If untreated, visual impairment can rapidly progress to severe consequences. In the proliferative diabetic retinopathy (PDR) stage, angiogenesis becomes more pronounced, and its harmful effects become more apparent 55. The newly formed blood vessels are fragile and prone to rupture and hemorrhage. This also leads to retinal edema, which exacerbates retinal ischemia and can result in severe outcomes, including substantial vision loss or complete blindness.

Diabetic macular edema (DME) is a severe complication of DR, impacting all stages and forms of the disease; angiogenesis contributes significantly to its progression 56. Optical coherence tomography (OCT) is used to classify DME into distinct patterns, such as diffuse macular edema, cystoid macular degeneration, and serous retinal detachment 57. Despite significant advancements in understanding DR, the precise mechanisms underlying its development remain multifaceted and complex, involving various disrupted metabolic processes and immune responses 58. The intricate interplay between these complex mechanisms and the detrimental effects of angiogenesis presents considerable challenges in the clinical management of DR.

Current therapeutic approaches for DR encompass glycemic control, topical administration, laser therapy, intravitreal medications, and vitrectomy. In recent years, researchers have made significant advances in understanding the molecular mechanisms of abnormal angiogenesis in DR, as well as potential therapeutic targets. Previous studies have shown that vascular endothelial growth factor (VEGF) plays a crucial role in promoting angiogenesis, while matrix metalloproteinase X1 (MMP-X1) is involved in early-stage vascular destruction and late-stage neovascularization in DR. Xinsheng Li *et al.* 59 have shown that the exosome lncRNA-MIAT/miR133a-3p/MMP-X1 axis may be a potential intervention for DR. Based on previous research and the correlation analysis in this study, exosome-delivered lncRNA-MIAT could jointly inhibit the expression of VEGF and MMP-X1 via miR-133a-3p, potentially suppressing abnormal angiogenesis in DR. Moreover, lncRNA-MIAT could modulate not only VEGF but also the expression of MMP-X1 through miR-133a-3p. Such findings deeply corroborate the role of lncRNA-MIAT in enhancing angiogenesis and suggest a new interventional and remedial target for DR.

**Figure 4:**
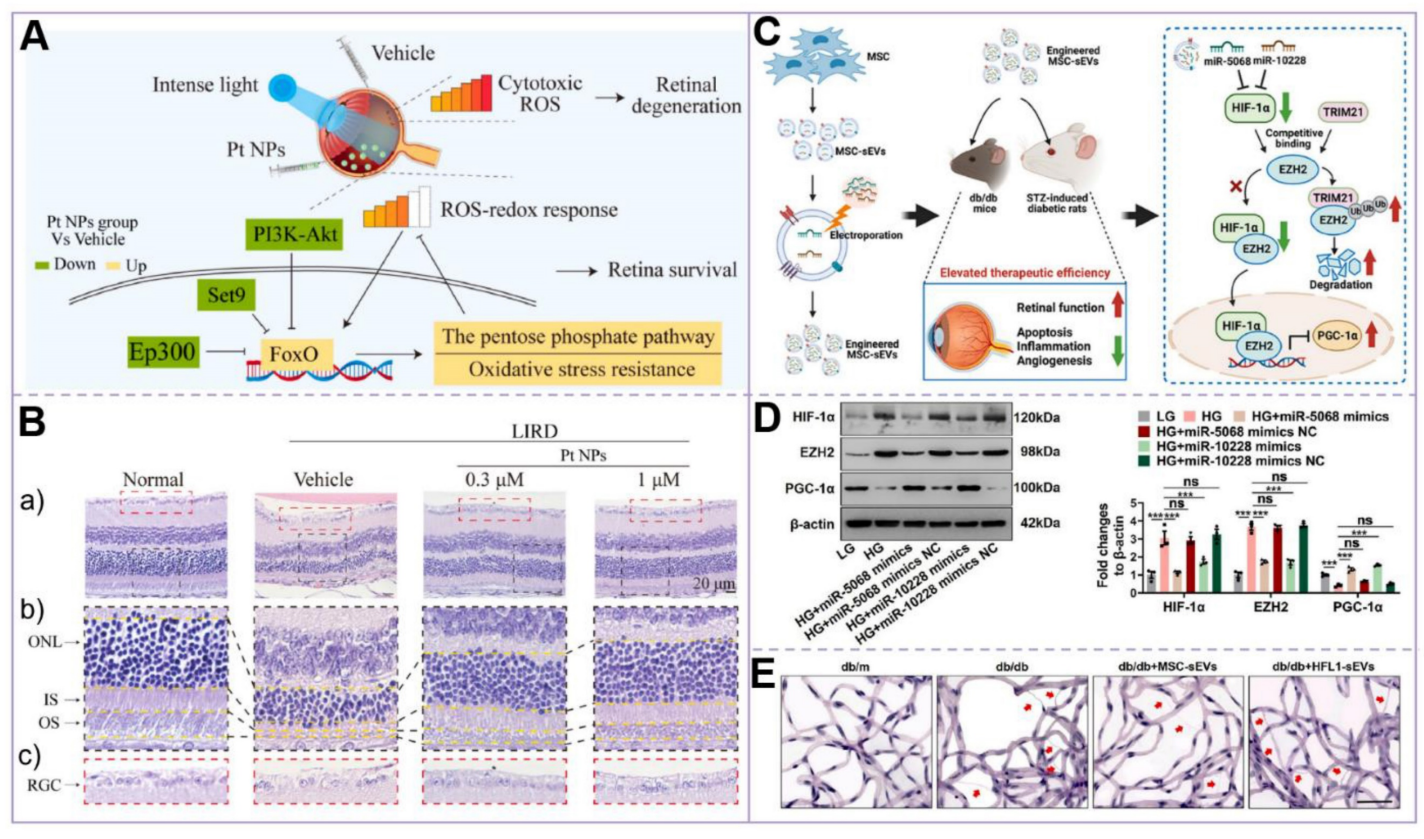
(A) Mechanism of Pt NPs in reducing ROS and protecting the retina from oxidative damage. In the Pt NPs-treated group, Pt NPs effectively reduced cytotoxic ROS levels and activated the FoxO signaling pathway, thereby regulating downstream protective mechanisms to mitigate oxidative damage and preserve retinal integrity; (B) H&E staining of the retina confirmed that Pt NPs effectively protected RPs from oxidative damage. (a) H&E staining images of each group of retinas. (b) An enlarged view of the area is in the black box in (a). (c) Enlarged view of the area in the red box in (a); (C) Mechanism of MSC-sEVs in treating diabetic retinopathy via the HIF-1α/EZH2/PGC-1α pathway. (D) Western blot analysis of HIF-1α, EZH2, and PGC-1α expression in retinal microvascular endothelial cells (RMECs) post-transfection; (E) Retinal trypsin digestion assay. Representative images of retinal vasculature following trypsin digestion, illustrating the structural changes in the retinal microvasculature. Scale bar: 50 μm. **(A-B)** Adapted with permission from 62, copyright 2023, Elsevier. **(C-E)** Adapted with permission from 63, copyright 2024, Ke Ai Publishing.

Similarly, Shaika Shanbagh *et al.* 60 found that the miR182-5p level in the eyes of PDR patients was relatively high. After transfecting retinal pigment epithelial (RPE) cells with the miR-182-5p mimic, they observed phenomena such as enhanced glycolysis and increased VEGF secretion, similar to those under high-glucose conditions. Therefore, regulating the expression of miR182-5p represents a potential method for achieving DR treatment. In addition, Tingyu Qin *et al.* 61 suggest that increasing the expression of polo-like kinase-3 (PLK-3) can also inhibit VEGF levels, thereby alleviating retinal vascular dysfunction in DR. In addition, researchers have made important breakthroughs in molecular mechanisms and cell therapy. On the one hand, the study by Lin Su *et al.* 62 demonstrated that platinum nanoparticles (Pt NPs) effectively reduce intracellular and retinal ROS and oxidative stress levels. Additionally, Pt NPs were shown to reduce photoreceptor apoptosis and maintain retinal structure and function in a DR-related light-induced retinal degeneration model. These findings provide a novel direction for DR intervention through the use of nanomaterials. On the other hand, Fengtian Sun *et al.* 63 successfully constructed engineered mesenchymal stem cell-derived small extracellular vesicles (MSC-sEVs), which enhanced their therapeutic efficiency in improving retinal function and reducing damage by increasing the levels of miR-5068 and miR-10228. These engineered vesicles exhibited a stronger therapeutic effect compared to natural MSC-sEVs, thereby opening a new avenue for the cell-free treatment of DR. These studies not only elucidate the complex pathological mechanisms of DR but also provide a scientific foundation for the development of novel therapeutic targets and strategies. In the future, therapeutic strategies integrating nanotechnology and extracellular vesicles are anticipated to emerge as a significant complementary approach for treating DR **(Figure 4)**.

### 2.3 Treatments for other common diabetic eye diseases

Among the various ophthalmic complications associated with DM, cataracts and glaucoma are also significant contributors to visual impairment. This section provides a concise overview of the treatment strategies for diabetic cataract (DC) and glaucoma.

#### 2.3.1 Therapeutic interventions for DC

DC is characterized by lens opacification, which not only threatens vision but may also exacerbate DR. Individuals with DM tend to develop cataracts earlier than those without DM 64. Surgical intervention remains the primary therapeutic approach for DC, though it may induce secondary complications such as diminished vision, vitreous hemorrhage, and choroidal detachment. Pharmacological strategies, including the use of aldose reductase inhibitors (e.g., Fadaprestat, Ranitidine), antioxidants (e.g., curcumin, vitamin C), and glycation inhibitors (e.g., ibuprofen, aspirin), are being explored 65. However, the efficacy of these treatments is often limited by the lipophilicity of certain drugs, which restricts their topical use. Clinical practice emphasizes strict glycemic control and continuous ophthalmic surveillance throughout the disease. Preoperative administration of NSAIDs in DC patients with NPDR or PDR may help mitigate postsurgical inflammatory responses 66.

#### 2.3.2 Glaucoma management in diabetic patients

Primary open-angle glaucoma (POAG) is the main form of glaucomatous neuropathy, marked by chronic, progressive loss of retinal ganglion cells and their axons, leading to irreversible visual field defects and potential blindness 67. Elevated intraocular pressure (IOP) and its fluctuations are major risk factors in the development and progression of POAG. Additional risk factors include advanced age, myopia, and variations in central corneal thickness. The association between DM and POAG is increasingly recognized, with epidemiological studies showing a higher incidence of POAG in individuals with DM compared to those without DM 68. DM and glaucoma share several common pathophysiological mechanisms, with DM known to induce hypertension. The primary treatment goal for POAG is to reduce IOP through pharmacological, laser, or surgical interventions. Furthermore, DM can exacerbate retinal injury in POAG through oxidative stress and the accumulation of AGEs, contributing to vascular dysfunction and retinal ischemia. A deeper understanding of these mechanisms is crucial for developing targeted therapies to address concurrent DM and POAG, potentially halting or slowing the progression of vision loss. The integration of advanced diagnostic tools and personalized approaches is critical for optimizing outcomes, given the complex interplay between DM and POAG 69.

## 3. Innovative nanotechnology: revolutionizing eye disease treatment

**Figure 5:**
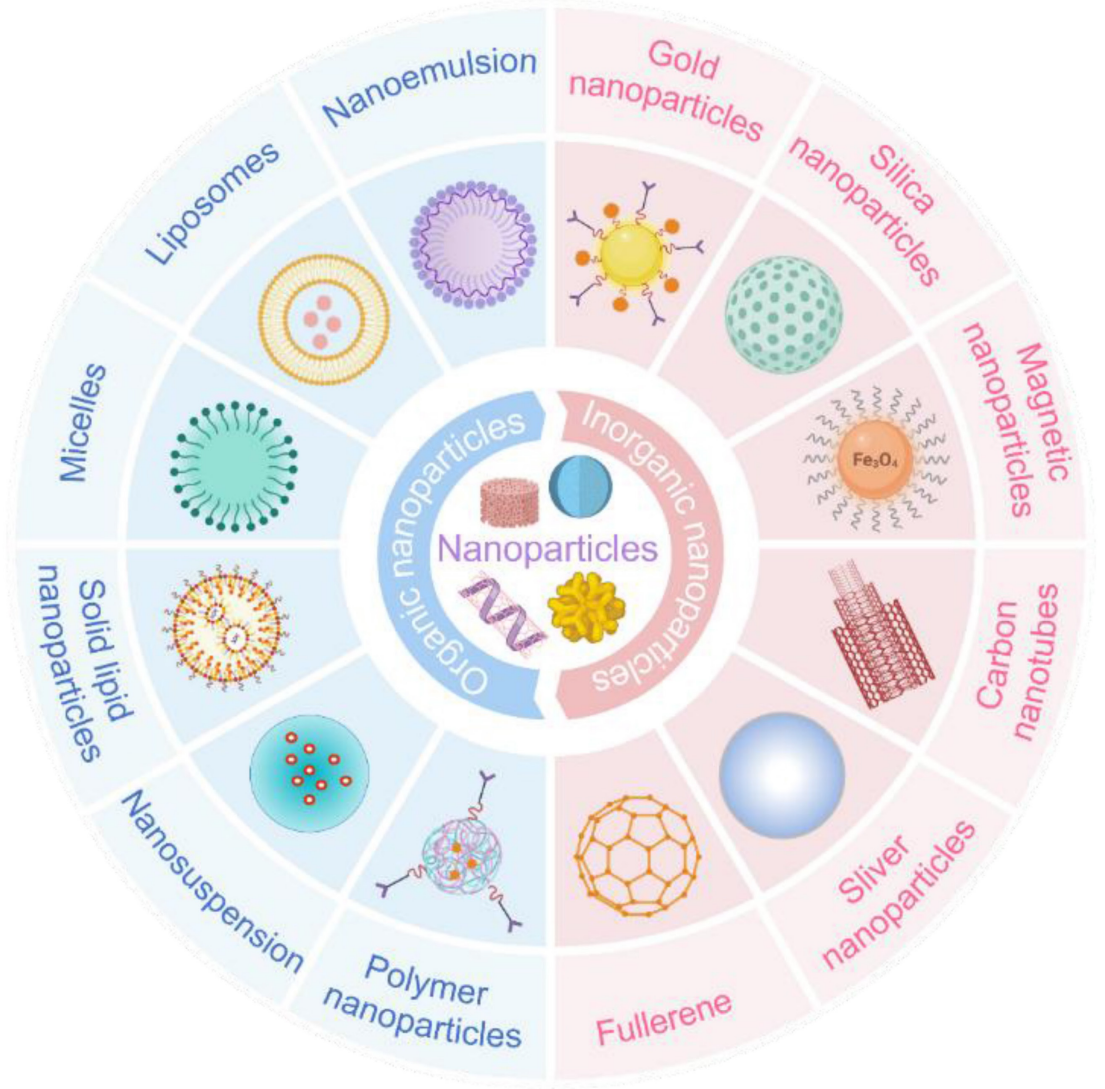
Some common nanoparticles, which is divided into two categories: organic nanoparticles and inorganic nanoparticles. Each type has its characteristics that make it suitable for specific applications. Created with BioRender.com. (http://biorender.com).

Nanotechnology is an emerging discipline focusing on the properties and applications of materials with structural dimensions ranging from 0.1 to 100 nm. It has been extensively applied across diverse fields, including environmental science and chemistry 70, 71. Nanoparticles address conventional drugs' limitations and offer novel disease surveillance strategies 72. Compared to traditional formulations, nano-preparations offer several advantages: their small particle size enables them to cross barriers inaccessible to conventional treatments; they exhibit targeted delivery, directing drugs precisely to the site of action; their sustained-release properties extend the drug's therapeutic duration; and they minimize drug degradation by enzymes in the body. Nanotechnology is currently employed in diverse routes of administration, including intravenous injection, oral, and topical administration, for treating a broad range of diseases including cancer, ocular inflammation, and diabetic complications 73 **(Figure 5)**.

The advent of nanotechnology has had a profound impact on various sectors of medicine, with ocular treatments experiencing notable advancements. Several marketed and approved nano-preparations for ocular treatments are listed in **Table 2**. The introduction of these products not only expands the range of ophthalmic therapies but also demonstrates the feasibility and potential of nanosystems in ocular applications, promising improved efficacy and safety in the treatment of eye diseases.

### 3.1 Core Functions of Nanotechnology in Eye Disease Therapy

**Figure 6:**
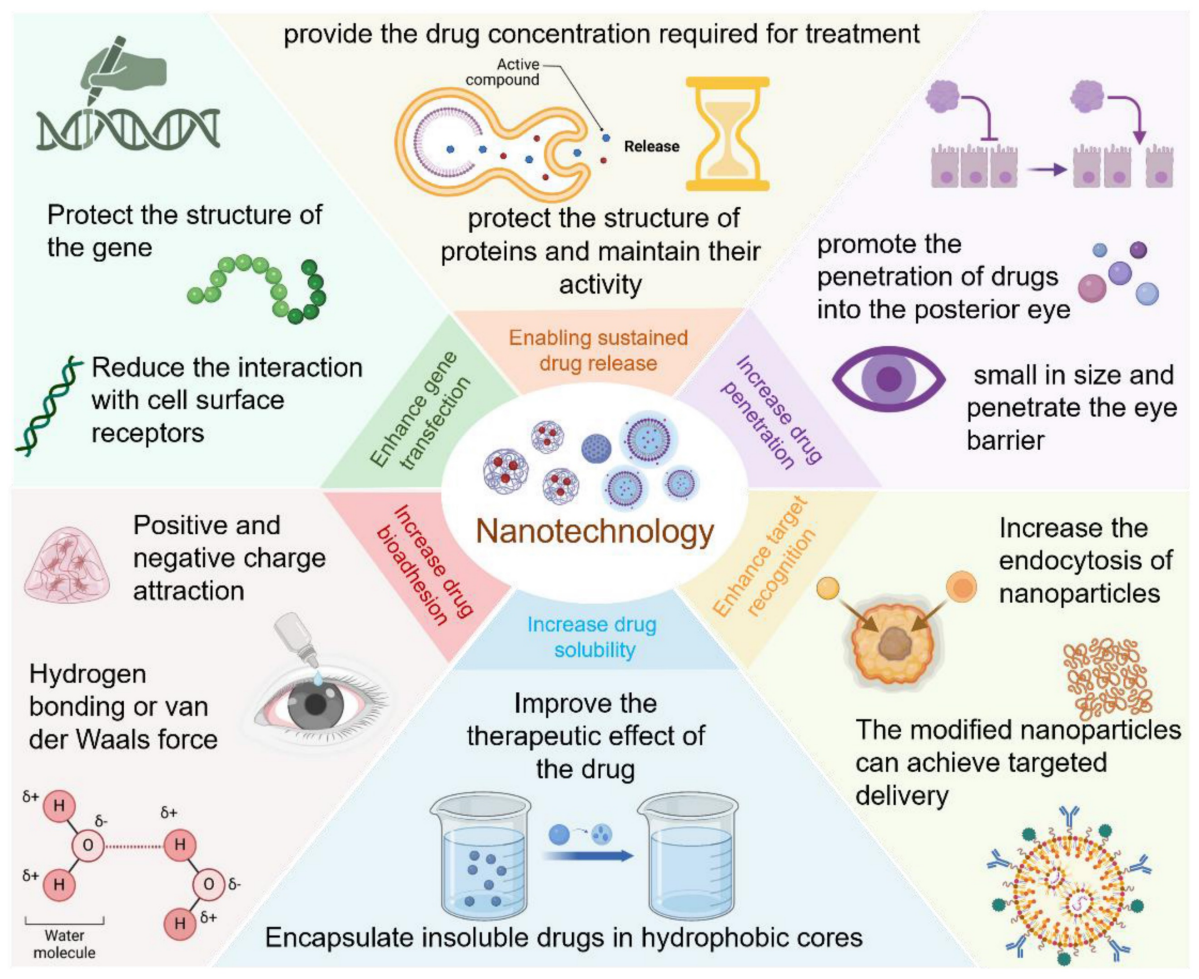
Summary of the applications of nanotechnology in eye treatment. Created with BioRender.com. (http://biorender.com).

The drug delivery systems for diabetic eye therapies face inherent limitations, particularly in drug solubility and ocular retention. Nanotechnology can address these issues, thereby enhancing treatment efficacy. In the exploration of novel treatments for eye diseases, the application of nanotechnology has significantly improved the delivery of macromolecules, including genes, to ocular tissues. Beyond its essential role in therapy, nanotechnology has also been employed in the detection and monitoring of diabetic eye conditions **(Figure 6)**.

#### 3.1.1 Enhancing drug solubility

The limited solubility of some approved drugs and most investigational agents restricts their clinical use 93. Many medications are formulated as suspensions due to poor solubility, but this form often leads to foreign body sensations and can obstruct lacrimal glands. Nanocarriers such as micelles can significantly enhance drug solubility. Micelles utilize their amphiphilic properties to encapsulate insoluble drugs in hydrophobic cores, forming transparent aqueous solutions. Triamcinolone acetonide, a widely used corticosteroid with anti-inflammatory effects, has been employed to treat diabetic eye diseases like DED, DR, and DME. Loading it into poly(ethylene glycol)-block-poly(ε-caprolactone) (PEG-b-PCL) micelles increased its solubility fivefold, while using poly(ethylene glycol)-block-poly(lactic acid) (PEG-b-PLA) micelles enhanced solubility tenfold 94. Alpha-lipoic acid (ALA), known for its antioxidant properties, has been investigated for diabetic keratopathy and retinopathy 95. In micelle preparations, its stability and solubility were significantly improved, with Soluplus® polymeric micelles increasing its solubility tenfold 96, 97.

Dapagliflozin (Dapa), a sodium-glucose cotransporter 2 (SGLT2) inhibitor used to therapy DM, has shown promise in targeting Epithelial-Mesenchymal Transition (EMT) biomarkers. This approach may reduce SGLT2 activation and related pathways in DC, while nanotechnology-based Dapa eye drops significantly improve Dapa's aqueous solubility, offering great potential for treating DC 98. Albumin's unique three-dimensional network structure offers significant advantages in drug delivery by efficiently encapsulating hydrophobic drugs and improving their solubility 99, 100. Nanoparticles formulated with albumin have been particularly effective in increasing the solubility and ocular bioavailability of curcumin, a treatment for DR, demonstrating that improved solubility contributes positively to therapeutic efficacy 101.

Furthermore, Siyu Gui *et al.* 102 successfully synthesized ultrasmall Fe-Quer nanozyme by coupling quercetin with low-toxicity iron ions. This innovative method not only effectively addressed the low solubility of quercetin but also endowed it with the ability to mimic three important antioxidant enzymes (superoxide dismutase, catalase, and peroxidase), thereby significantly enhancing its capacity to scavenge ROS. This dual enhancement in structure and function has endowed Fe-Quer nanozyme with significant potential in preventing and delaying the development and progression of DR. Strategies to enhance drug solubility, particularly through the development and optimization of nanoemulsions, have also demonstrated considerable potential. For the treatment of DED, ibuprofen has been formulated using chitosan-coated nanoemulsions to improve its solubility and stability 103. The incorporation of chitosan imparts a positive charge to the nanoemulsions, thereby enhancing their interaction with the negatively charged ocular surface mucins, prolonging the residence time of the drug on the ocular surface, and improving its bioavailability.

#### 3.1.2 Improving drug permeability

One of the major challenges in ocular drug delivery systems is the low bioavailability of drugs. This is mainly because most ophthalmic drugs are administered on the eye's surface, and special barriers and structural features limit drug absorption and distribution 104. Ocular barriers include the dilution and rapid turnover of tears, the barrier effect of the corneal epithelium, and the blood-retinal barrier 105. Therefore, the development of novel drugs capable of entering the eye's posterior segment is crucial for the effective treatment of ocular diseases.

Pioglitazone, a thiazolidinedione antidiabetic drug for T2DM treatment, increases the body's insulin sensitivity by activating peroxisome proliferator-activated receptor γ (PPAR-γ), thus helping to lower blood glucose levels. Moreover, PPAR-γ receptors are also implicated in DR 106, 107. Umesh D. Laddha *et al.* prepared pioglitazone-loaded PLGA nanoparticles for ocular drug delivery via a single-emulsion solvent evaporation method. The surface modification was achieved by enhancing the interaction with the eye using the non-ionic surfactant sorbate 80, initially attaining the goal of drug delivery to the eye's posterior segment 108.

A research team from Tianjin Medical University has developed a novel liposome-based permeable eyedrop formulation (pDrops) utilizing liposomal technology 109. This innovative delivery system consists of a core-shell structure, with the therapeutic agent encapsulated in the liposome core and chitosan forming the outer shell. The chitosan coating improves the adhesion of pDrops to mucin in tears, thereby extending the drug residence time on the ocular surface. Upon contact with the anterior ocular surface, pDrops transiently open tight junctions between corneal and conjunctival epithelial cells, facilitating drug penetration into the posterior segment and overcoming ocular barriers. Notably, pDrops can simultaneously deliver both hydrophobic curcumin and hydrophilic ganciclovir to the posterior segment of the eye.

As research progresses, nanozymes have drawn increasing attention. Nanozymes are nanomaterials with inherent enzyme-like properties and can achieve self-cascade catalysis that is difficult for enzymes or small molecules to achieve. Min Tian *et al.* noted the important role of metal nanoparticles in DR treatment and designed an eye drop based on ultra-small copper nanodots nanozymes capable of penetrating into the fundus 110, 111. This eye drop adopts a comprehensive microenvironment regulation mode based on the nanoenzyme cascade, which has multiple functions, including alleviating hypoxia, scavenging free radicals, and anti-inflammation.

#### 3.1.3 Augmenting pharmaceutical compound bioadhesion

Local administration, typically via eye drops, has remained the primary method for treating eye diseases. However, the rapid turnover of the tear film and nasolacrimal drainage significantly reduces the retention of eye drops, with only a small portion reaching the posterior segment of the eye. Nanotechnology offers a solution by promoting the rapid adhesion of drugs to the ocular epidermis tissue, thereby extending their retention time. The primary mechanisms underlying nanoparticle-mucosal adhesion include (1) Diffusion, where polymer chains penetrate the mucosal layer; (2) Adsorption, involving interactions between the carrier's functional groups and mucin through hydrogen bonding or van der Waals forces; and (3) Electrostatic attraction between positively and negatively charged components of the carrier and mucin 112-114.

Although the complete pathogenesis of DED remains unclear, ocular surface inflammation caused by bacteria and fungi, as well as hyperosmotic stress of the tear film, which leads to the excessive production of ROS, are considered to be the basic pathological changes of DED. Excessive accumulation of ROS can lead to not only oxidative stress on the ocular surface but also pathological changes in ocular tissues, such as reduced mucin production and immune dysregulation. Compared with traditional ocular antioxidants, nanozymes possess certain advantages 115. Haoyu Zou *et al.* 116 developed a cerium oxide nanoenzyme, capable of effectively scavenging excessive ROS produced by oxidative damage and hypertonic stimulation. They exploited the affinity between phenylboronic acid (PBA) and o-diols to dynamically bind the tear film mucin layer, which contains many neighboring diol structures, to PBA fragments, thereby greatly improving the ocular surface adhesion force of the nanoenzyme.

To enhance the therapeutic effect in glaucoma, Yingshan Zhao *et al.* 117 developed a novel nano-delivery system capable of penetrating the mucin layer and delivering drugs to the cornea through electrostatic interactions between cationic carriers and anionic mucin in the tear film. Unlike simple drug molecular solutions, the nanosystem rapidly interacts with mucin upon contact with the ocular surface. This interaction not only prevents metabolism by tear and corneal enzymes but also enables controlled drug release, thereby prolonging the retention time on the ocular surface. Furthermore, nano-micelles have shown significant potential in ophthalmology. Sun Xingchen *et al.* 118 designed and synthesized a phenylboronic acid-conjugated chitosan-vitamin E copolymer for loading voriconazole, which treats fungal keratitis, to form mucin-adhesive nanomicelles. They also demonstrated these nanomicelles' strong permeability and ocular surface retention capabilities.

Similarly, Haijie Han *et al.* 119 developed a cabozantinib-loaded nanoparticle carrier, Cabo-NPs, based on cationic peptides. This carrier achieves mucoadhesion through the interaction between cationic peptides and mucosa, thereby prolonging the drug retention time on the cornea and enhancing bioavailability. It effectively inhibits corneal neovascularization (CNV), with efficacy comparable to dexamethasone but without significant side effects, offering a safer alternative for CNV treatment. The bioadhesive glycoconjugated nanoplatform developed by Yanlong Zhang *et al.* 120 has also advanced the treatment of CNV. This nanoplatform is self-assembled from amphiphilic boronic acid-based copolymers, demonstrating good stability and biocompatibility. It specifically binds to corneal epithelial cells via the boronic acid module, achieving long-term retention on the corneal surface. Furthermore, it releases dexamethasone (DEX) in a controllable manner, enabling efficient transcorneal drug delivery.

#### 3.1.4 Optimizing nanomedicine surface for better targeting and uptake

Interactions between specific receptors and nanoparticles can lead to enhanced endocytosis, thereby increasing cellular uptake and drug efficacy 121. This principle has been effectively utilized in the development of targeted drug delivery systems 122. For instance, hyaluronic acid (HA), a negatively charged polysaccharide, binds to CD44 receptors on various retinal cells, facilitating the internalization of nanoparticles through HA-mediated endocytosis. Research demonstrates that HA-coated nanoparticles significantly improve retinal targeting compared to uncoated counterparts, emphasizing the potential of HA coatings for the localized therapy of posterior segment ocular disorders 123. Folic acid (FA) receptors are expressed in the cell layer of the RPE 124, prompting studies to explore FA-modified nanoparticles for targeted drug delivery to the retina 125. Dave V. and colleagues modified gold nanoparticles with synthetic FA-b-PEG block copolymer to facilitate the delivery of sorafenib tosylate for treating DR and reducing neovascularization. The observed reduction in retinal tortuosity and vascular dilation post-treatment with nanoparticles demonstrated the efficacy of FA-b-PEG-modified nanoparticles in targeted delivery 126. These strategies demonstrate the potential of receptor-targeted nanoparticles in enhancing the delivery of drugs to ocular tissues, offering new avenues for the management of retinal disorders.

Liyang Zhou *et al.* 127 constructed a novel photosynthetic hybrid system (Cyano@Au@Ir) by utilizing cyanobacteria as a nanozyme carrier loaded with gold and iridium nanoparticles, thereby optimizing the surface of nano-drug formulations. In contrast to traditional nanoparticles modified with polysaccharides or small molecules, cyanobacteria leverage the light-transmitting properties of the eye to continuously produce oxygen in the retinal region under illumination. This process creates a favorable metabolic environment and enhances targeting specificity. The micrometer-sized Cyano@Au@Ir, when administered via subretinal injection, is distributed precisely between the RPE and photoreceptor cells. This delivery method circumvents rapid metabolism in the eye, enabling the system to exert its effects over an extended period in the retinal area. Li-Jyuan Luo *et al.* 128 functionalized chitosan and ZM241385 (a non-xanthine adenosine receptor antagonist) on the surface of hollow cerium nanoparticles (hCe NPs), enabling them to open tight junctions in the corneal epithelium and target the ciliary body. Additionally, they utilized the antioxidant and anti-inflammatory properties of hCe NPs to achieve multi-target treatment for glaucoma.

#### 3.1.5 Sustained drug release mechanisms

The primary treatment for posterior segment diseases caused by DM is intravitreal injection, but frequent injections elevate the risk and reduce patient compliance. To maintain therapeutic concentration while minimizing the number of invasive administrations, a drug delivery system capable of sustained drug release in the posterior eye is essential. Nano-drug delivery systems for curing posterior eye disorders have been extensively studied, as nanomedicines can remain in the vitreous for extended periods, ensuring the required drug concentration for treatment.

Research has shown that cerium (Ce) effectively eliminates free radicals and peroxides, reducing lipid peroxidation. Mesoporous silica nanoparticles containing cerium (CeCl_3_@mSiO_2_) have demonstrated efficacy in reducing oxidative stress in lens epithelial cells 129. In further studies, these nanoparticles exhibited sustained and controlled drug release for at least 60 hours. When injected intraperitoneally into a DC rat model, results indicated that CeCl_3_@mSiO_2_ nanoparticles improved disease progression by reducing oxidative damage 130.

Maintaining effective doses of protein drugs for posterior ocular diseases is more challenging due to their structural instability, susceptibility to protease degradation, and short half-lives. Nanocarriers not only delay drug release but also protect protein structure and maintain activity. Anti-VEGF drugs, which are high-molecular-weight proteins, prevent new blood vessel formation by blocking VEGF from binding to its receptor 131. Bevacizumab is approved for treating ocular diseases caused by neovascularization 132, but its short half-life and the requirement for continuous injections result in side effects and financial burdens 133.

Encapsulating bevacizumab in mesoporous silica nanoparticles extends its release up to 28 days *in vitro*, and results in a longer mean retention time (MRT) *in vivo*, compared to the free drug. This approach prolongs the drug's half-life while maintaining its biological activity, as confirmed by its anti-angiogenic efficacy 134. Rong X *et al.* developed a dual-controlled sustained-release system by incorporating insulin-loaded chitosan nanoparticles into a thermo-responsive hydrogel. Insulin release from individual nanoparticles lasted only 1 day, but the sustained release from nanoparticles within the hydrogel extended beyond 60 days 135. A mucoadhesive and responsive nanogel serves as a carrier for the sustained delivery of timolol in glaucoma therapy. Animal experiments demonstrated that a single administration can reduce and maintain IOP in rabbits at normal levels for over 48 hours 136. To enhance the therapeutic efficacy of the hydrophobic drug lutein, researchers developed a chitosan-sodium alginate-fatty acid nano-carrier system. This system exhibits sustained-release properties, which significantly improve lutein bioavailability in conditions like DR and DC 137.

#### 3.1.6 Boosting transfection efficiency and prolonging expression duration

Compared to conventional drugs, gene therapy has the potential to address the underlying causes of diseases, thereby eliminating the need for repeated administration 138. The cornea and retina are considered ideal targets for ocular gene therapy 139. Ocular gene therapy's transfection efficiency is hindered by poor gene permeability, nuclease degradation, and cytoskeletal barriers. Following the approval of Luxturna, the first FDA-approved recombinant adeno-associated virus gene therapy, research into ocular gene therapy, particularly for diabetic eye diseases, has gained significant momentum 140. However, the limited encapsulation capacity of viral vectors often raises safety concerns, particularly regarding their inherent immunogenicity. In contrast, non-viral vector nanoparticles provide a safer, more cost-effective, and non-toxic alternative, with simpler production processes. In response to elevated ROS levels in CNV, Anqi Liu *et al.* 141 developed lipid nanoparticles containing TK bonds. These nanoparticles degrade in response to the intracellular ROS environment, enabling the effective release of siRNA that silences VEGF expression, offering a novel therapeutic strategy for CNV.

**Figure 7:**
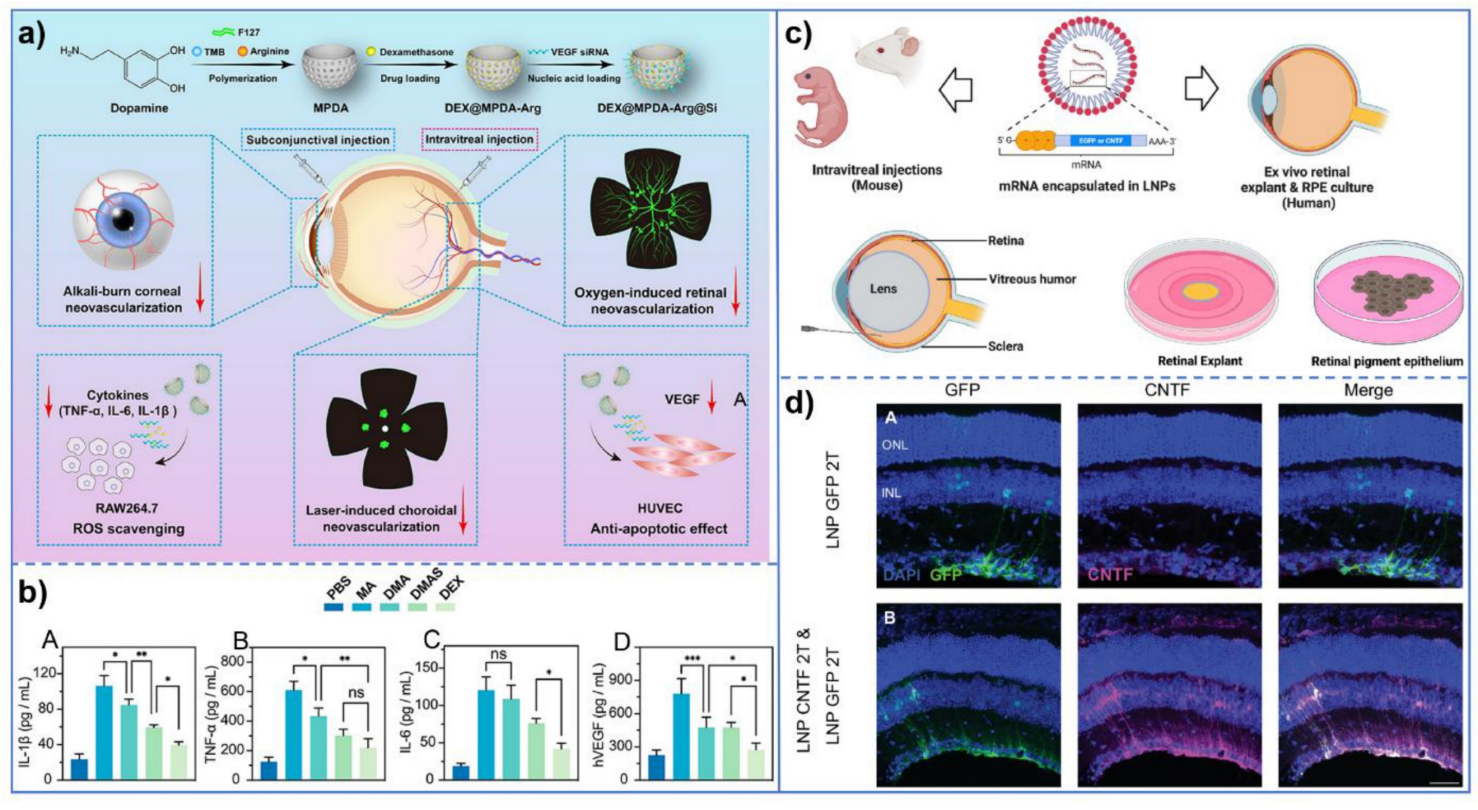
(a) Schematic illustration of DEX@MPDA-Arg@Si (DMAS) for ocular neovascular disease (OND) therapy. The diagram outlines the preparation process of MPDA-based nanomedicine, including polymerization, drug loading, and functionalization. Additionally, it demonstrates the therapeutic efficacy of DMAS in multiple OND models, such as alkali burn-induced corneal neovascularization (CoNV), oxygen-induced retinopathy (OIR), and laser-induced CNV; (b) Expression levels of pro-inflammatory cytokines in RAW264.7 cell supernatant. Quantification of IL-1β (A), TNF-α (B), and IL-6 (C) levels in the supernatant of RAW264.7 cells. Data are presented as mean±SD (n=3); (c) Lipid nanoparticle delivery of mRNA into the mouse or human retina; (d) Transfection of CNTF mRNA into mouse Müller glial cells using 2T LNP. CNTF mRNA encapsulated in 2T lipid nanoparticles was successfully transfected into mouse Müller glial cells, as evidenced by the specific expression of CNTF in these cells, confirming efficient delivery into retinal tissue. **(a-b)** Adapted with permission from 143, copyright 2024, DOVE Medical Press. **(c-d)** Adapted with permission from 144, copyright 2024, Association for Research in Vision and Ophthalmology.

In the realm of retinopathy, retinopathy of prematurity (ROP) presents its distinct pathological features, with pathological retinal neovascularization and inflammatory reaction being the predominant aspects. In light of this pathological characteristic of ROP, Keke Huang *et al.* 142 developed folate-chitosan-modified nanomaterials for the delivery of miR-223. These nanomaterials can induce the transformation of retinal microglia from a pro-inflammatory phenotype to an anti-inflammatory phenotype, thereby achieving the treatment of ROP via anti-inflammatory and anti-angiogenesis pathways. It is noteworthy that the findings of this study are not confined to the treatment of ROP; rather, its broader significance lies in opening up a new avenue for the treatment of ocular inflammation-neovascularization-related diseases and providing a novel and potential treatment strategy. Furthermore, Xiaochen Ma *et al.* 143 developed a nanomedicine system by integrating VEGF-siRNA, dexamethasone, and bioactive mesoporous polydopamine (MPDA) to target multiple pathological mechanisms of ocular neovascular diseases through synergistic drug actions. This nanomedicine system not only inhibits angiogenesis but also exerts anti-inflammatory effects via dexamethasone and leverages the antioxidant properties of MPDA. In the context of gene delivery, Cheri Z. Chambers *et al.* 144 employed lipid nanoparticles (LNPs) as non-viral vectors for mRNA delivery. Compared to traditional adeno-associated virus (AAV) vectors, LNPs exhibit lower immunogenicity and can deliver larger transgenes, offering a novel approach for ocular gene therapy. These studies not only enhance the transfection efficiency of gene therapy but also prolong gene expression duration, thereby advancing novel strategies and methodologies for ocular gene therapy **(Figure 7)**.

In general, nanoparticles offer several key advantages in gene delivery, including (1) protection of gene structure from enzymatic degradation; (2) enhancing gene delivery and increasing cellular uptake compared to single gene therapies; (3) minimizing interactions with cell surface receptors and reducing off-target effects of siRNA; and (4) sustaining intracellular gene delivery. Nanocarriers enhance drug solubility, penetration efficiency, and cellular internalization while sustaining gene delivery, offering more effective treatments for diabetic ocular complications. The nanoparticles developed in one study exhibit the synergistic advantages described above, leading to improved therapeutic outcomes. **Table 3** presents an overview of the nanoparticles referenced in the text.

### 3.2 Broader impacts of nanotechnology on eye disease management

Nanotechnology not only addresses challenges in drug delivery for diabetic ocular complications but also enables early monitoring and non-invasive detection. Persistent hyperglycemia in eye tissue leads to severe complications, making early glucose monitoring essential to prevent these outcomes. Portable glucose monitoring devices require finger-pricking, causing discomfort and low compliance 157. As an alternative, Google and Novartis developed glucose-sensing contact lenses for DM diagnosis, though these lenses require power, limiting their convenience 158. Park S *et al.* 159 developed colorimetric contact lenses that detect glucose based on the color shift between cerium's oxidation states (Ce^3+^ to Ce^4+^). Cerium oxide nanoparticles (CNP) were conjugated with glucose oxidase (GOx) using PEG, forming CNP-PEG-GOx nanocomposites, which were integrated into a (hydroxyethyl) methacrylate solution to create the lenses. The hydrogen peroxide generated from glucose oxidation by GOx converts colorless Ce^3+^ to yellow Ce^4+^, and a smartphone-based algorithm is used to analyze the color intensity for glucose quantification.

The method detected glucose in tears from rabbit and human eyes, providing a reliable, simple, non-invasive way to monitor glucose levels. Schauval *et al.* encapsulated anti-VEGF drugs with VEGF aptamer-functionalized carbon dots, using the inherent fluorescence of carbon dots to achieve non-invasive detection of intraocular drug concentrations 160. In addition, Dong Yun Lee *et al.* 161 developed a clinically viable suction cup (SD) strip biosensor, which incorporates a sensing paper coated with CNP and GOx. The sensor collects tear fluid through non-invasive contact with the lower eyelid conjunctiva (IPC), thereby avoiding eye irritation. It is designed for single-use and short-term application, enabling convenient self-monitoring of blood glucose levels.

The application of nanotechnology in the management of eye diseases has demonstrated significant potential in enhancing drug delivery and therapeutic efficacy. For instance, epigallocatechin gallate-conjugated gold nanoparticles (E-Au NPs) can convert light energy into thermal energy under near-infrared (NIR) irradiation, thereby achieving mild photothermal therapeutic effects 162. This photothermal transformation property allows E-Au NPs to elevate the temperature at the infection site, disrupt bacterial cell structures, interfere with their physiological processes, and ultimately enhance bacterial lethality. Notably, E-Au NPs exhibit a pronounced inhibitory effect on drug-resistant bacteria, including methicillin-resistant Staphylococcus aureus (MRSA). In contrast to traditional high-temperature photothermal therapy, this mild photothermal approach minimizes damage to surrounding normal tissues and is particularly suitable for temperature-sensitive regions such as the eyes. In the treatment of ocular infections, such as keratitis, the combination of E-Au NPs and near-infrared irradiation not only effectively controls infection but also reduces inflammatory responses and promotes corneal tissue repair. This highlights the significant potential of integrating nanotechnology with photothermal therapy in ocular therapeutics.

Nanotechnology has also advanced the field of molecular imaging, particularly for tracking Vascular Cell Adhesion Molecule-1 (VCAM-1), a key inflammatory marker in DR progression. Although several imaging methods have been developed to visualize VCAM-1, many still suffer from limitations such as insufficient sensitivity and invasiveness 163, 164. Uddin M D I *et al.* 165 developed gold nanoparticles functionalized with VCAM-1-targeted antisense hairpin DNA. These nanoparticles can hybridize with VCAM-1 mRNA in cells and induce fluorescence. The nanoprobe allows for the specific imaging of VCAM-1 mRNA in TNF-α-activated mouse retinal microvascular endothelial cells (MRMECs), without the need for transfection reagents or cell permeabilization. This innovation represents a significant advancement in non-invasive monitoring techniques for DR.

Early detection, early intervention, and early treatment have always been the key criteria for the treatment of ocular complications. Therefore, its early diagnosis, especially that of DR, is of great significance in clinical practice. Long-term hyperglycemia in diabetic patients gradually causes retinal microvascular damage, and the early lesions may not present obvious symptoms. If DR can be diagnosed early, timely intervention measures, such as controlling blood glucose, blood pressure, and blood lipids and other risk factors, can be taken to delay the development of lesions. Moreover, early diagnosis provides the best opportunity for more targeted treatment, such as laser therapy and anti-VEGF therapy. These treatments can achieve better therapeutic effects and reduce the risk of serious consequences, such as vision loss. Therefore, to achieve efficient DR detection, many researchers have explored from different perspectives. Among them, Vadanasundari *et al.* developed vanadium core-shell nanorods loaded with vanadium oxide on carbon dioxide. These nanorods can detect metabolic changes related to DR and effectively evaluate disease progression, and the related research has been advanced to the clinical trial stage 165. In addition, Hainsworth D P *et al.* 166 developed a colorimetric nanotechnology-based paper sensor for measuring 8-Hydroxy-2'-Deoxyguanosine, a biomarker of DR, in urine, which facilitates convenient and accurate home-based testing.

In recent years, the rapid development of nanoprobe technology has enabled a further improvement in the accuracy and sensitivity of DR detection. Nanoprobes possess unique physical and chemical properties, such as extremely small size and customizable surface functionalization. These properties enable the nanoprobes to specifically identify biomarkers or cellular changes related to DR. For example, Yuanlin Zhang *et al.* 167 used vascular endothelial growth factor receptor 2 (VEGFR-2) as a biomarker for early DR diagnosis and developed a high-brightness adhesive fluorescent nanoprobe with biodegradable materials. This nanoprobe not only overcomes the obstacle of insufficient brightness but also promotes cellular immune response research in DM. Similarly, Linjie Wang *et al.* 168 designed a multifunctional nanoreactor based on Ru nanoparticles, which integrates biological and nanoenzymes and serves as a nanoprobe, thereby developing a new colorimetric/smartphone integrated sensing platform. Unlike the previous nanoprobe studies for DR, this nanoreactor primarily focuses on the critical link of blood glucose detection. By integrating the characteristics of biological enzymes and nanoenzymes, the nanoprobe can efficiently achieve rapid and accurate detection of blood glucose under near-neutral pH conditions. With the assistance of the smartphone integrated sensing platform, this detection method offers the advantages of convenience, low cost, and ease of promotion, providing a new technical means for the daily monitoring of DM. Simultaneously, this also further showcases the versatility and immense potential of nanotechnology in DM-related research. Whether for the diagnosis of diabetic complications or the direct detection of blood glucose, nanotechnology offers diversified solutions to enhance the health management of patients with diabetic eye complications.

## 4. Conclusion and Future Perspectives

The rising incidence of DM has increasingly focused attention on the management and prevention of its associated complications. Among these complications, ocular issues arising from chronic hyperglycemia are the leading causes of vision loss and blindness in individuals with DM. Current treatments for DR and other ocular conditions often require frequent dosing or invasive procedures such as intravitreal injections. This regimen not only burdens patients but also leads to suboptimal ocular bioavailability due to the eye's protective barriers. Consequently, there is a pressing need for advanced drug delivery systems capable of enhancing the efficacy and safety of ophthalmic therapies for DM.

Nanotechnology has emerged as a promising solution to these challenges. Nanocarriers offer significant advantages in ocular drug delivery, including improved solubility of poorly water-soluble drugs, extended ocular epidermis residence time, and improved drug permeability across eye tissues. These properties make them especially suitable for targeting the posterior segment of the eye, where many diabetic complications originate. Additionally, the ability of nanocarriers to encapsulate macromolecules, such as antibodies or genetic material, presents new opportunities for gene therapy in treating DM-related ocular diseases.

The purpose of gene therapy is to correct genetic defects or modify abnormal physiological processes, thereby providing longer-lasting benefits than conventional drug treatments. In the context of diabetic eye disorders, these therapies hold substantial promise due to their ability to target specific sites and provide sustained therapeutic effects. Nanocarriers can deliver therapeutic genes directly to diseased ocular tissues, minimizing systemic exposure and reducing side effects. This precise delivery method is expected to significantly benefit patients by reducing treatment frequency and improving adherence to therapy.

Despite the promising potential of nanotechnology in ocular treatments, several specific challenges remain. A major concern is the safety profile of nanoparticles, particularly their potential toxicity and immune responses in ocular tissues. Improved methods for tracking and monitoring nanoparticle distribution are also needed to ensure targeted delivery without damaging healthy tissues. In addition, the scalability and cost-effectiveness of nanoparticle-based formulations must be improved to facilitate widespread adoption. Current regulatory frameworks may not fully accommodate the unique properties of nanomedicines, presenting challenges for product approval and commercialization. Future research should focus on developing safe and efficient nanoparticles, establishing standardized preclinical assessment protocols, and collaborating with regulatory authorities to develop appropriate regulations for nanomedicine.

In conclusion, the future of nanotechnology in ophthalmology is promising. Advances in materials science have enabled the development of biocompatible and degradable nanoparticles that reduce toxicity and enhance drug delivery efficiency. These smart delivery systems can be engineered to respond to environmental triggers, such as pH or temperature, releasing their therapeutic payload precisely where needed. Additionally, nanotechnology offers the potential for combination therapies, where multiple drugs are loaded onto a single nanocarrier, enhancing treatment outcomes for complex conditions like age-related macular degeneration or glaucoma. As ongoing research addresses current limitations and regulatory agencies establish guidelines for the safe use of nanomedicines, nanotechnology is poised to play a transformative role in ophthalmology, offering patients more effective treatments with fewer side effects.

## Figures and Tables

**Figure 8:**
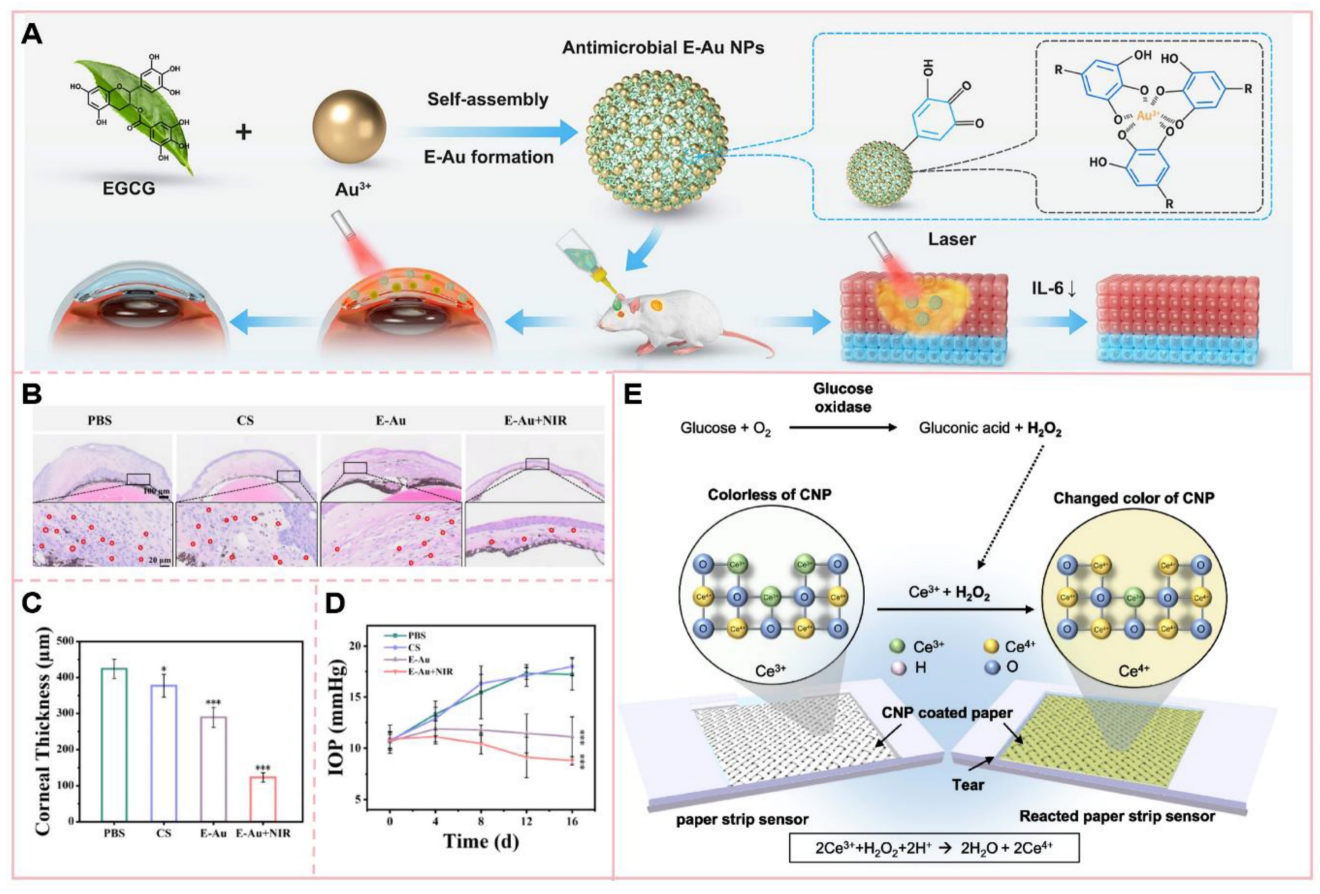
(A) Epigallocatechin gallate (EGCG)-functionalized gold nanoparticles (E-Au NPs) were synthesized via a facile one-step self-assembly method, demonstrating potent antibacterial and antibiofilm properties against [specific pathogens, e.g., Gram-positive and Gram-negative bacteria; (B) H&E staining of the cornea and (C) quantitative analysis of corneal thickness; (D) Intraocular pressure measurement; (E) Schematic illustration of the reaction mechanism on the sensing paper immobilized with CNP and GOx for glucose detection. The colorimetric detection is based on the oxidation of Ce³⁺ (colorless) to Ce⁴⁺ (yellow) induced by glucose concentration in tears, resulting in visible color changes on the indicator paper. **(A-D)** Adapted with permission from 162, copyright 2024, BioMed Central. **(E)** Adapted with permission from 161, copyright 2023, American Association for the Advancement of Science.

**Table 1:** Clinical and experimental treatment strategies for DM-related corneal diseases.

Treatment strategy	Therapeutic agent	Advantages	Limitation	Ref.
Blood glucose control	Nateglinide; Glibenclamide; Exenatide; Pioglitazone	Increase epithelial wound healing; Inhibit changes in Descemet	Prone to hypoglycemia	23, 24
	Insulin eye drops/ implants	Accelerate corneal wound healing; prevent subbasal corneal nerve loss	Increase levels of VEGF and worsening DR	25-27
Lipid control	Fibrates	Anti-inflammatory action; Enhance corneal innervation and sensitivity	Side effects of T2D patients	28
	Statins	Significantly lower lipid peroxidation in diabetic neuropathy patients	Controversial effects	29, 30
Opioid antagonists	Naltrexone	Corneal epithelial wound healing	Autoxidation	31, 32
Ergoline derivatives	Megerin	Promote corneal wound healing and improve corneal sensitivity	Current research has limitations	33, 34
Antioxidant	β-carotene, α-lipoic acid	Promote corneal epithelial regeneration	Eye transport difficulties	35
Aldose reductase inhibitor	Ranistat; CT-112; Kinostat^®^	Faster epithelial wound healing	the clinical effect is controversial	36, 37
Surgery	Corneal Transplantation	One-time treatment of corneal defect	Low success rate; Prone to corneal infection; Possible rejection	38
Treatment strategy	Therapeutic agent	Advantages	Limitation	Ref
Lubricant	Artificial tears	Relieve eye dryness	Unable to boost tear production	39
Anti-inflammatory	Corticosteroid	Reduce inflammation levels of dry eye; Prevention of corneal epithelial injury	Cause infections; Induce cataracts; Cannot long-term use	40-42
	Nonsteroidal Anti-inflammatory Drugs (NSAIDs)	Avoid steroid side effects, relieve dryness, heal corneas, protect goblet cell	Cannot improve the production of tears; Reduce corneal sensitivity	43, 44
	Cyclosporine A	Improve tear production	Poor ocular drug availability	45
	Tacrolimus	Anti-inflammatory; Restore tear production; Reduce dry eye symptoms	Ocular toxicity; Dose dependence	46
	Autoserum	Safe and effective; Facilitates wound healing in diabetic patients	Induce secondary infections; specific causative factors are doubt	47-49
Growth factor	NGF; SP; C-peptide	Increase neurite growth and subbasal nerve density; Promote epithelial wound healing; Restore corneal sensitivity	Limited sources; High cost; Serious side effects; Short biological half-life; The mechanism is not clear;	50
Gene therapy	mRNA; siRNA	Improve corneal wound healing rate; Promote corneal nerve regeneration	Too much target	51
Fatty acids	Docosahexaenoic acid (DHA)	Antioxidative stress	High dose of toxic side effects	52, 53

**Table 2:** Some marketed/approved nano-preparations for the treatment of ocular diseases.

Formulation	Active Ingredient	Product	Main composition	Administration Method	Adaptive Disease	Ref.
Nanoemulsion	Cyclosporin A	Restasis^®^	Castor oil; Polysorbate 80; Carbomer	Eye drop	DED	74, 75
	Difluprednate	Durezol^®^	Castor oil; Polysorbate 80	Eye drop	Eye inflammation	
	Cyclosporin A	Ikervis^®^	Medium chain triglycerides; Poloxamer 188	Eye drop	DED	76
Micelle	Cyclosporin A	Cequa^®^	HCO-40; OC-40	Eye drop	DED/ KCS	77, 78
Liposome	Vitamin A palmitate; Vitamin E	Lacrisek^®^	Hydrogenated phospholipids	Eye drop	DED	79
	Hyaluronic acid	Tears Again^®^	Liposomal soy lecithin; Phenoxyethanol	Eye spray	DED	
	Verteporfin	Visudyne^®^	Unsaturated EPG; DMPC	Intravitreal injection	AMD	80, 81
Nanoparticle	Pegaptanib sodium	Macugen^®^	PLGA	Intravitreal injection	Neovascularization; AMD	82, 83
Implant	Fluocinolone acetonide	Retisert	PVA	Intravitreal implant	Uveitis	84
	Dexamethasone	Ozurdex	PLGA	Intravitreal implant	Uveitis	
	Ganciclovir	Vitrasert^®^	PVA; EVA	Intravitreal injection	AIDS-associated cytomegalovirus retinitis	85
	Fluocinolone acetonide	Iluvien^®^	Polyimide	Intravitreal injection	DME	
		Yutiq^®^	Polyimide	Intravitreal injection	Chronic non-infectious uveitis	
Suspension	Triamcinolone acetonide	Trivaris	Sodium hyaluronate; Sodium phosphate	Intravitreal injection	Uveitis	86, 87
		Triesence	Sodium carboxymethyl cellulose; Polysorbate 80	Intravitreal injection	Diabetic macular edema	
		Dexycu^®^	Acetyl triethyl citrate	Intravitreal injection	Inflammation	85
	Bromfenac	Bromsite^®^	Bromfenac durasite; Synthetic polymer of cross-linked polyacrylic acid	Topical administration	Inflammation; Pain after cataract surgery	88
	Betaxolol	Betoptic S^®^	Ion exchange resins	Topical administration	Glaucoma	89
	Indomethacin	Indocollirio^®^	Hydroxypropyl-b-cyclodextrin	Topical administration	Eye inflammation	90
	Besifloxacin	Besivance^®^	Polycarbophil	Eye drop	Infection	91
Hydrogel	Timolol maleate	TIMOPTIC-XE^®^	Gelrite	Eye drop	Glaucoma	92

Dry eye disease (DED); Polyoxy hydrogenated castor oil (HCO-40); Octoxynol-40 (OC-40); Keratoconjunctivitis sicca (KCS); Age-related macular degeneration (AMD); Unsaturated Egg Phosphatidylglycerol (EPG); Dimyristoyl phosphatidylcholine (DMPC); Poly (lactic-co-glycolic acid) (PLGA); Polyvinyl alcohol (PVA); Ethylene vinyl acetate (EVA); Diabetic Macular Edema (DME).

**Table 3:** The above-mentioned nanoparticles for drug and gene delivery.

Therapeutic Agent	Composition Of Recipe Type	Disease Type	Experimental Model	Size	Mechanism Or Principle	Ref.
Glycyrrhizin; Genistein	Micelles	Diabetic Corneal Wound Healing	C57BL/ 6J Mice	29.50±2.05 Nm	Small Size Enhances Corneal Penetration	145
Triamcinolone Acetonide	Micelles	Inflammation	New Zealand Albino Rabbits	146.90±4.29 Nm	Mucus Adhesion	94
	Nanoparticle	DR	Sprague-Dawley Rats	184±2 Nm	Mucosal Adhesive	146
Pt Nanocluster	Nanoparticle	DC	Sprague-Dawley Rats	160 Nm	Mucosal Adhesion; Penetration Enhancement	147
Pioglitazone	Nanoparticle	DR	Wistar Rats	171.7 Nm (PLGA 50:50)	Penetration Of Nanoparticles	147
Apatinib	Nanoparticle	DR	Wistar Rats	222.2±3.56 Nm	Enhance Target Recognition; Mucus Adhesion	123
Cyclosporin-A	Nanogel	Dry Eye	New Zealand Albino Rabbits	15.8±0.26 Nm	Mucosal Adhesive	148
Curcumin	Nanogel	Multiple Eye Diseases	New Zealand White Rabbits	221.2 Nm	Increased Solubility; Extended Residence Time	149
Cecl_3_	Nanoparticle	DC	Wistar Rats	87.6±8.9nm	Sustained Release; Enable Controlled Release of Drugs	150
Gold Nanoparticles; Sorafenib Tosylate	Nanoparticle	DR	Chinchilla Rabbits	50.93 Nm	Sustained Release	126
Sirna	Lipoplexes	DR	Sprague⿿Dawley Rats	\	Enhancement Of Gene Transfection Ability	151
Sirna	Nanoparticle	Choroidal Neovascularization	Pigmented Rats	186±4.3 Nm	Reduce The Enzymatic Degradation	152
Insulin	Nanogel	DR	Sprague-Dawley Rats	137.5 Nm	Controlled Release	135
ALA	Micelle	DM-Related Corneal Diseases	Bovine Cornea	84.7 Nm	Increase Solubility	153
Corticosteroid Drugs	Micelle	Retinal Degenerative Diseases	Human RPE Cells	<500 Nm	Mucosal Adhesion Properties; Permeability Enhancement	154
Aons	Nanoparticle	Diabetic Corneal Wound Healing	Organ Cultured Human Diabetic Cornea; Lecs	\	Enhance Gene Transfection	155
Curcumin	Nanogel	Multiple Eye Disease	Hcecs	15.51±0.15 Nm	Increased Penetration	156
